# On the Action of Iodide of Iron upon Phthisis

**Published:** 1861-03

**Authors:** 

**Affiliations:** Physician to the Hospital for Consumption at Brompton


*On the Action of Iodide of Iron upon Phthisis.* By Dr. COTTON, Physician to the Hospital for Consumption at Brompton.—Dr. Cotton tests the action of this compound upon twenty-five cases, not selected, but taken just as they arrived at the hospital, those only being excluded which were either at too advanced a stage to admit of any remedial treatment, or which happened to be laboring under some inflammatory or other important complication.

The iodide was administered in the form of the syrupus ferri iodidi mixed with water, in doses of a drachm, twice and sometimes three times a day. It was continued, according to its effects, for various periods; the shortest one month, and the longest three months.

Of the twenty-five patients, eleven were males, and fourteen females; their ages varied from eighteeu to forty years. Eight were in the first stage of the disease; three were in the second stage; and fourteen presented positive evidence of pulmonary cavities. In ten cases there was great improvement; in four, moderate improvement; and in eleven, no improvement.

In analyzing these resnlts, it was found that of the ten greatly improved, four were in the first stage of the disease, and six in the third. Of the four moderately improved, one was in the first stage, one in the second, and two in the third. Of the fourteen in whom no improvement was noticeable, three were in the first stage, two in the second, and nine in the third.

Three cases of improvement were decidedly marked; two of these patients, whose disease was only in the first stage, left the hospital with their pulmonary affection quiescent, and apparently restored to health, calling themselves indeed quite well; and the other, although more advanced, and in the third stage of phthisis, was marvellously improved, and able to resume his occupation.

In two cases, hæmoptysis came on during the administration of the iodide, and in two the iodide was discontinued on account of headache and dyspepsia. The spitting of blood probably was in no way attributable to its use, hæmoptysis having occurred previously in the same patients; but the other symptoms having ceased or diminished with a change of medicine, might perhaps be fairly referred to its employment. Except in those instances, the iodide of iron appeared to agree very well with the patients, several of whom improved very much in appetite and strength.

Three of the patients in whom there had been no improvement, afterwards derived benefit from other medicines.

In eight of the cases cod liver oil was occasionally taken in combination with the iodide; one-half of these we found to belong to the class of improved, the other half to that of not improved.

Of the fourteen improved cases, ten gained in weight, some of them very considerably, three remained in *statu quo*, and one lost two pounds while under treatment. The improvement, however, was not always in proportion to the increase of weight, some of the patients who had increased the most, having improved the least.

It is, perhaps, worth recording, that one of the patients took, accidentally, an ounce of the syrup, which would contain thirty-two grains of the iodide of iron. It produced a distressing feeling of weight in the epigastrium, attended with nausea and subsequent purging. For several weeks afterwards, there was a loss of appetite, depression of spirits, and a constant feeling of uneasiness in the region of the stomach, all of which, however, gradually passed away, leaving the patient apparently none the worse for the overdose.

After making due allowance for the influence of concomitant circumstances, such as rest, hygiene, and hope, all of which claim a prominent share in the improvement of all hospital patients, we are justified, Dr. Cotton thinks, in arriving at the following conclusions:

1. Syrup of the iodide of iron, in doses of a drachm, twice or three times a day, occasionally produces headache, with some dyspeptic symptoms; but for the most part, it is found to agree very well with consumptive patients.

2. Although very far from exhibiting what might be termed a *specific* effect, it nevertheless seems to act very beneficially in a fair number of consumptive cases, especially in those where the disease is only at an early stage.

3. Under its influence the patient's weight is generally increased.—*Med. Times and Gaz.*

